# Analysis of Vaccine Effectiveness Against COVID-19 and the Emergence of Delta and Other Variants of Concern in Utah

**DOI:** 10.1001/jamanetworkopen.2021.40906

**Published:** 2021-12-23

**Authors:** Lindsay T. Keegan, Shaun Truelove, Justin Lessler

**Affiliations:** 1 Division of Epidemiology, Department of Internal Medicine, University of Utah, Salt Lake City, Utah; 2 International Vaccine Access Center, Department of International Health, Johns Hopkins Bloomberg School of Public Health, Baltimore, Maryland; 3 Department of Epidemiology, University of North Carolina at Chapel Hill

## Abstract

This cross-sectional study analyzes the effectiveness of COVID-19 vaccines against Delta and other variants in Utah.

## Introduction

Since the emergence of SARS-CoV-2, vaccines have been heralded as the best way to curtail the COVID-19 pandemic. Clinical trials have shown SARS-CoV-2 vaccines to be highly efficacious against both disease and infection.^1^ However, those vaccines currently in use were primarily tested against early lineages. Data on vaccine effectiveness against variants of concern (VOCs) remains limited, including the Delta variant (B.1.617.2).

The spread of VOCs with significantly higher transmissibility (eg, the Delta variant), has raised concerns about ability for vaccines to sustainably control SARS-CoV-2. These concerns are exacerbated by the increasing number of cases reported in fully vaccinated individuals as the Delta variant spreads globally, potentially signaling a decreased vaccine effectiveness for VOCs.^2^ A preliminary study^3^ in the UK on the vaccine effectiveness of the BNT162b2 (Pfizer/BioNTech) and ChAdOx1 (AstraZeneca) vaccines found a 6% reduction in efficacy for the BNT162b2 vaccine and a 12% reduction for the ChAdOx1 vaccine. However, because of Delta’s recent emergence and low case numbers, this study only included 27 fully vaccinated cases altogether.

## Methods

This cross-sectional study uses the Strengthening the Reporting of Observational Studies in Epidemiology (STROBE) reporting guideline. This work was based on aggregate publicly available data and hence deemed not to be human participant research nor to require informed consent, per the Common Rule.

The Utah Department of Health (UDOH) began monitoring VOCs in October 2020 and first identified 2 cases with Delta in mid-April 2021.^4^ In this analysis, we use publicly available data from UDOH,^4^ including unlinked daily numbers of cases alongside their vaccination status and proportion of variant among cases. Currently, UDOH sequences 10% of reported cases and collects vaccination coverage for all cases.

**Figure 1.**
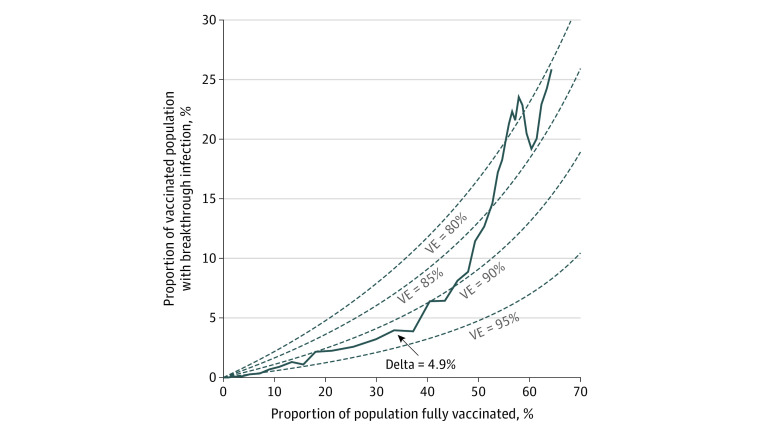
Daily Percentage of Breakthrough Cases and Population That is Fully Vaccinated The 14-day moving mean of the percentage of breakthrough cases against the percentage of the population vaccinated (blue) from January 16 to October 9, 2021, with the theoretical curves for the expected percentage of breakthrough cases with vaccine effectiveness ranging from 80% to 95% (gray dashed lines).

**Figure 2.**
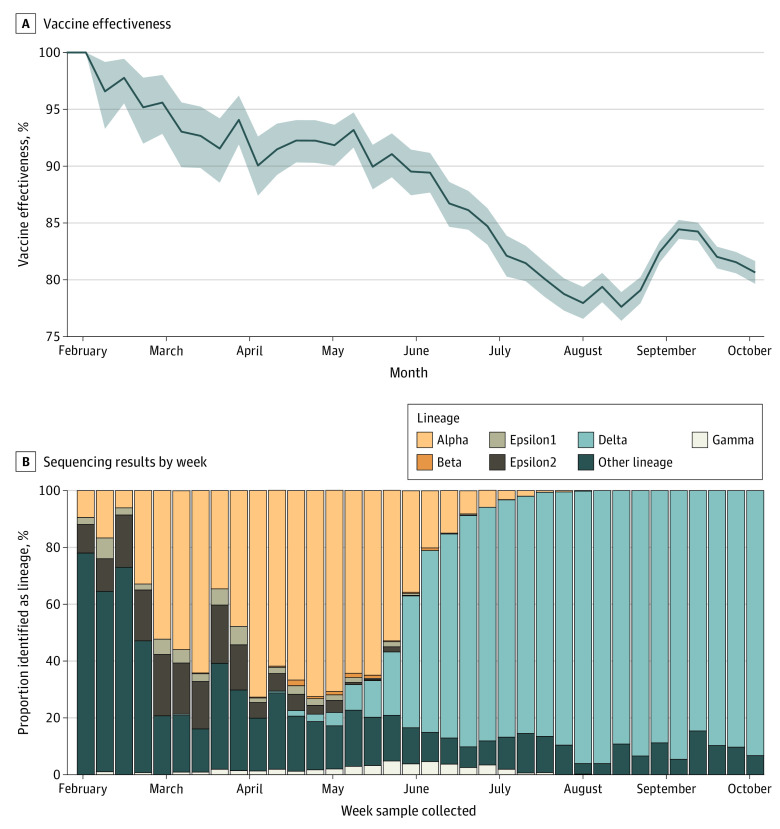
Daily Vaccine Effectiveness Against SARS-CoV-2, and Sequencing Results by Week in Utah A, The 14-day moving mean of vaccine effectiveness against SARS-CoV-2 from January 16 to October 9, 2021, and the 95% CI (shaded gray region). B, The weekly percentage of all sequenced samples by lineage from the week starting on January 17, 2021, to the week starting on October 3, 2021.

The percent of breakthrough cases in Utah was increasing at a concerning rate (Figure 1) with the emergence and spread of Delta (Figure 2). To determine the effectiveness of the vaccines deployed in Utah against testing positive to SARS-CoV-2, we estimated the combined daily vaccine effectiveness (VE_t_) using the field evaluation approach in Orenstein et al.^5^ We then extend this method to understand the rate of breakthrough infections in the face of a rapidly changing variant landscape. To estimate the vaccine effectiveness against Delta alone, we partition VE_t_ into the vaccine effectiveness of the Delta variant (VE_δ_) and the vaccine effectiveness of all other variants (VE_1_–_δ_) such that VE_t_ = VE_1_–_δ _ × P_1_–_δ _+ VE_δ _ × P_δ_, with P_δ_ as the proportion of sequenced cases that were from the Delta variant, and P_1- δ _as the proportion sequenced that is not Delta.

R version 3.6.2 (R Project for Statistical Computing) was used for calculations, and data analysis was performed from January to October 2021. No statistical testing was conducted as part of this analysis.

## Results

As of October 18, 2021, 1 726 946 of 3 205 958 Utahans (66.6%) of the eligible population were fully vaccinated, which was defined as more than 14 days after the final vaccine dose; 982 938 (57.1%) with the BNT162b2 vaccine, 592 801 (34.4%) with the mRNA-1273 (Moderna) vaccine, and 147 193 (8.53%) with the JNJ-78436735 (Johnson & Johnson) vaccine.^4^ The proportion of breakthrough cases started increasing faster than expected given population vaccination rates in mid-May (Figure 1), reaching 79 of 542 cases (14.6%) by the end of June vs 35 (6.4%) of the population as expected with a 90% effective vaccine. We estimated the effective vaccine effectiveness from all vaccination in Utah from January 16 to October 15, 2021 (Figure 2A) and found that vaccine effectiveness declined from 90% (95% CI, 88%-92%) in mid-May to a low of 78% (95% CI, 76%-79%) in mid-August before rebounding to 81% (95% CI, 80%-82%) by October (Figure 2A). This decline occurred simultaneously as a rapid increase in the proportion of cases infected with the Delta variant (Figure 2B). Since the first case of Delta was detected, Delta has rapidly outcompeted all other variants, and, as of October 3, 2021, it represents 93.3% of all SARS-CoV-2 viruses sequenced in Utah (Figure 2B). If we attribute the entire change in vaccine effectiveness to the Delta variant (ie, VE_t_ = VE_1_–_δ_  × P_1_–_δ_ + VE_δ_  × P*_δ_*), the estimated vaccine effectiveness against Delta would be 81.2% (95% CI, 80.8%-81.6%).

## Discussion

Our results suggest a modest reduction in vaccine effectiveness against COVID-19 in Utah associated with the expansion of the Delta lineage in the state. This reduction in the effectiveness of available vaccines associated with the arrival of novel VOCs, rather than waning immunity, is concerning. These should serve as a caution throughout the US that the Delta variant can bring renewed outbreaks, even in highly vaccinated populations. If there is a consistent trend of increasing immune escape as new variants arise, it could eventually undermine the effectiveness of current vaccines and necessitate mass revaccination.

This study was limited. We used publicly available data that was collected for outbreak surveillance. The breakthrough case definition allows for self-reporting of vaccination status, resulting in fully vaccinated individuals reporting at higher rates (ie, 10% overreporting of vaccination would bring the vaccine effectiveness to 83.8%). Also, only 10% to15% of cases were sequenced each day, prioritizing breakthrough cases and outbreaks, potentially causing an oversampling of Delta variants. Furthermore, there is a delay of up to 2 weeks in reporting sequence data; therefore, estimates of breakthrough infection by variant are lagged by 2 weeks. Additionally, we do not account for known demographic differences (eg, urbanicity) in unvaccinated and breakthrough infections. While the association between trends seen in Delta and the observed vaccine effectiveness is consistent with reductions in vaccine effectiveness being related to Delta's emergence, we cannot rule out the possibility of the waning of vaccine-induced immunity playing some role with the available data.
